# Sleep patterns and cardiovascular disease risk in US participants: a comprehensive analysis

**DOI:** 10.3389/fnins.2024.1447543

**Published:** 2025-01-09

**Authors:** Yue Wu, Zhizheng Li, Peng Zhao, Jiajing Xu, Min Yuan

**Affiliations:** ^1^Department of Cardiovascular Medicine, People's Hospital of Xiangxi Tujia and Miao Autonomous Prefecture, The First Affiliated Hospital of Jishou University, Jishou, China; ^2^Department of Neurology, Zixi Hospital of Jiangxi Provincial People’s Hospital, Zixi County People’s Hospital, Fuzhou, China; ^3^Department of Neurology, Jiangxi Provincial People’s Hospital, The First Affiliated Hospital of Nanchang Medical College, Nanchang, China

**Keywords:** sleep disorders, trouble sleeping, cardiovascular disease, association, clinical epidemiology

## Abstract

**Background and purpose:**

To evaluate the association between sleep-related factors, including sleep duration, self-reported sleep disturbances, and diagnosed sleep disorders, and the risk of cardiovascular disease (CVD) in US participants.

**Methods:**

The data of this study from the National Health and Nutrition Examination Survey (NHANES) conducted between 2007 and 2014. Sleep factors were assessed using a standardized questionnaire, and overall sleep scores were calculated on a scale of 0 to 3. The participants were classified into three sleep pattern groups: poor sleep pattern (overall sleep score ≤ 1), intermediate sleep pattern (overall sleep score = 2), and healthy sleep pattern (overall sleep score = 3). CVD was defined based on self-reported questionnaire responses. Logistic regression models were used to investigate the association between sleep factors and CVD.

**Results:**

Among 21,115 participants, 2,245 (10.6%) were diagnosed with CVD. Participants with poor sleep patterns had a significantly higher risk of CVD (OR = 1.82, 95% CI: 1.52–2.16, *p* < 0.001). Self-reported trouble sleeping (OR = 1.53, 95% CI: 1.32–1.78, *p* < 0.001), and sleep disorder (OR = 2.09, 95% CI: 1.75–2.50, p < 0.001) were related to an increased risk of CVD. However, no such association was observed for either short (OR = 1.12, 95% CI: 0.95–1.33, *p* = 0.174) or long sleep durations (OR = 1.14, 95% CI: 0.90–1.45, *p* = 0.266). Our study also suggested an interaction between sleep patterns and age (P for interaction = 0.002).

**Conclusion:**

This study highlights the significant association between poor sleep patterns and an increased risk of CVD in US participants.

## 1 Introduction

Cardiovascular disease (CVD), which includes congestive heart failure (CHF), coronary heart disease (CHD), angina, myocardial infarction (MI), and stroke, continues to be the primary reason for mortality and disease burden in numerous countries around the world (Joseph et al., 2017). Over the last decade, the global death toll from CVD has risen by 12.5%, contributing to almost one-third of all worldwide deaths (GBD 2015 Disease and Injury Incidence and Prevalence Collaborators, 2016). The exact causes of CVD are complex and involve an interplay of genetic and environmental factors. The predominant share of CVD cases can be mitigated by addressing modifiable behavioral risk factors, including poor sleep patterns, tobacco use, unhealthy dietary habits, obesity, and physical inactivity. Therefore, timely recognition of CVD is crucial to initiate prevention and control measures, commencing with counseling and addressing risk factors (WHO, 2021).

Sleep behaviors represent a vital metric, serving as a pivotal indicator of physiological health. Individual sleep behaviors typically exhibit interrelated patterns in a compensatory manner (Li et al., 2021; Fan et al., 2020). Human sleep patterns are crucial for evaluating general health and functionality. Sleep plays a critical role in modulating cardiovascular function, affecting the development of CVD in both the physiological and pathological states. Prior research (Saz-Lara et al., 2022; Miller and Howarth, 2023) have demonstrated that sleep can have a profound impact on the human autonomic nervous system, vascular tone, cardiac function, endothelial function, and coagulation. These effects correlate with alterations in sympathetic nerve activity (Panza et al., 1991), plasma catecholamine levels, and blood flow shear stress.

In most previous studies, sleep behaviors were examined in isolation, neglecting the intricacies and interrelationships among sleep parameters. There is a paucity of studies that have comprehensively examined sleep behaviors collectively. Investigations indicate that the combination of insufficient sleep duration and other adverse sleep behaviors, such as difficulty falling asleep and sleep disorders, increases the likelihood of developing CVD.

Therefore, using the NHANES database, we separately analyzed the relationships between sleep duration, sleep difficulties, sleep disorders, and CVD risk. We also defined the combination of these three factors as “sleep patterns,” which were also evaluated in relation to CVD risk.

## 2 Materials and methods

### 2.1 Data sources and study population

This cross-sectional study utilized data from NHANES, which was collected during the 2007–2008, 2009–2010, 2011–2012, and 2013–2014 cycles by the Centers for Disease Control and Prevention. NHANES was designed in a stratified, multistage probability sample survey to assess health and nutrition statistics. This survey is known for its nationally representative nature. The ethical clearance for NHANES was obtained from the NCHS Ethics Review Committee, and all participants provided written informed consent before their involvement.

Our study exclusively included individuals aged ≥18 years who had undergone an interview. Exclusion criteria comprised individuals with missing data related to cardiovascular disease, sleep factors, or covariates. This research followed the guidelines established by the Strengthening the Reporting of Observational Studies in Epidemiology (STROBE).

### 2.2 Definition of CVD

Our outcome is whether diagnosed with CVD. CVD is defined as at least one diagnosis of CHF, CHD, angina, MI, or stroke; the hard criteria include MI and stroke (Cai et al., 2020). We used a participant’s responses to the medical conditions questionnaire to determine whether they had CVD: “Has a doctor or other health professional ever told you had angina/heart attack/stroke/ CHD?” (Zhang et al., 2023).

### 2.3 Sleep factors and sleep pattern

Data regarding sleep duration were collected by asking the question, “How much sleep do you typically get on weekdays or workdays?” The duration was then classified into three categories: short (less than 7 h per night), normal (7–9 h per night), or long (more than 9 h per night), with 7–9 h per night serving as the reference group. To assess trouble sleeping and sleep disorders, responses to questions such as “Have you ever told a doctor or other health professional that you have trouble sleeping?” and “Have you ever been told by a doctor or other health professional that you have a sleep disorder?” were analyzed, respectively. To determine a comprehensive sleep score, several aspects of sleep were taken into account, including sleep duration, self-reported sleep difficulty, and diagnosed sleep disorders. A low-risk sleep score was assigned to individuals who slept between 7 and 9 h each night without experiencing any trouble sleeping or being diagnosed with a sleep disorder. A binary categorization was assigned to each sleep factor, with a low risk being indicated by 1 and a high risk being indicated by 0. The overall sleep patterns were determined to be either healthy, with an overall sleep score of 3, intermediate, with an overall sleep score of 2, or poor, with an overall sleep score ranging from 0 to 1. This resulted in a cumulative score that could range from 0 to 3 (Li and Shang, 2021).

### 2.4 Other covariates

This data was acquired through a computer-assisted automated approach, employing the U.S. Department of Agriculture (USDA) automated multiple-pass method to accommodate day-to-day variations. According to prior research (Fan et al., 2020; Saz-Lara et al., 2022; Kizilkilic et al., 2023), we evaluated various potential confounding factors, which included age, sex, race, education level, marital status, insurance, BMI (body mass index), hypertension, DM (diabetes mellitus), smoking status, physical activity, HbA1c, TC (total cholesterol), and HDL-C (high-density lipoprotein). Our analysis focused on these factors, as they have been identified as important in previous studies. The classification of race or ethnicity encompasses Mexican-American, other Hispanic, non-Hispanic White, non-Hispanic Black, and other races. Based on the U.S. education system and previous literature, the education level was divided into three categories: less than 9 years, 9–11 years, and more than 12 years (Hamad et al., 2019; Gurgel et al., 2021; Fleming et al., 2021; Liu et al., 2022). Married, never married, and others make up the different categories of marital status. Covered by health insurance (any insurance, no). BMI is calculated by dividing an individual’s weight in kilograms by their height squared (kg/m2). Hypertension is diagnosed when a person’s systolic blood pressure is 140 mmHg or higher and/or their diastolic blood pressure is 90 mmHg or higher, or if they have a prior diagnosis of high blood pressure (Xiao and Xiao, 2023). DM was determined on the basis of a questionnaire question about whether the patient had a history of diabetes. The smoking status of individuals was categorized into three categories, namely never, former, and current smokers, based on their responses to two questions from a questionnaire. The questions were: “Have you smoked at least 100 cigarettes in your entire life?” and “Do you currently smoke cigarettes?” Drinking habits were defined as having at least 12 alcoholic drinks per year (Yang et al., 2024). Physical activity was classified into three levels: sedentary, moderate, or vigorous (Liu et al., 2022).

### 2.5 Statistical analysis

In accordance with the guidelines set by NHANES for analysis, this study took into account the complexities of sampling designs and the associated weights (Johnson et al., 2013). The sample weight for the four NHANES cycles was determined by taking the initial two-year sample weight, dividing it by four, and subsequently assigning this adjusted weight to each individual participant. Furthermore, the variable denoting masked variance pseudo-PSU (SDMVPSU) is used to identify the primary sampling units (PSU), while the variable indicating masked variance pseudo-stratum (SDMVSTRA) is responsible for identifying the strata from which the PSUs are chosen.

Participants were categorized by sleep pattern for baseline characteristics. The normal distribution of continuous variables was evaluated using the Shapiro–Wilk statistical test, while differences in both continuous and categorical variables were investigated through independent t-tests and chi-squared tests. Logistic regression was employed to determine the relationship between each sleep factor and CVD by computing odds ratios (ORs) and 95% confidence intervals (95% CIs). In separate analyses, the connection between sleep patterns and CVD was explored.

Prior to conducting statistical power estimates, no *a priori* determination was made regarding the sample size, as it was solely based on the available data. The statistical software packages used for all analyses were R 4.2.2 (http://www.R-project.org, The R Foundation, Shanghai, China) (accessed on November 24, 2023) and Free Statistics software version 1.9. All participants underwent a descriptive study. The two-tailed test yielded a *p*-value of less than 0.05, indicating a significant result.

## 3 Results

### 3.1 Baseline characteristics of participants

**Figure 1:**
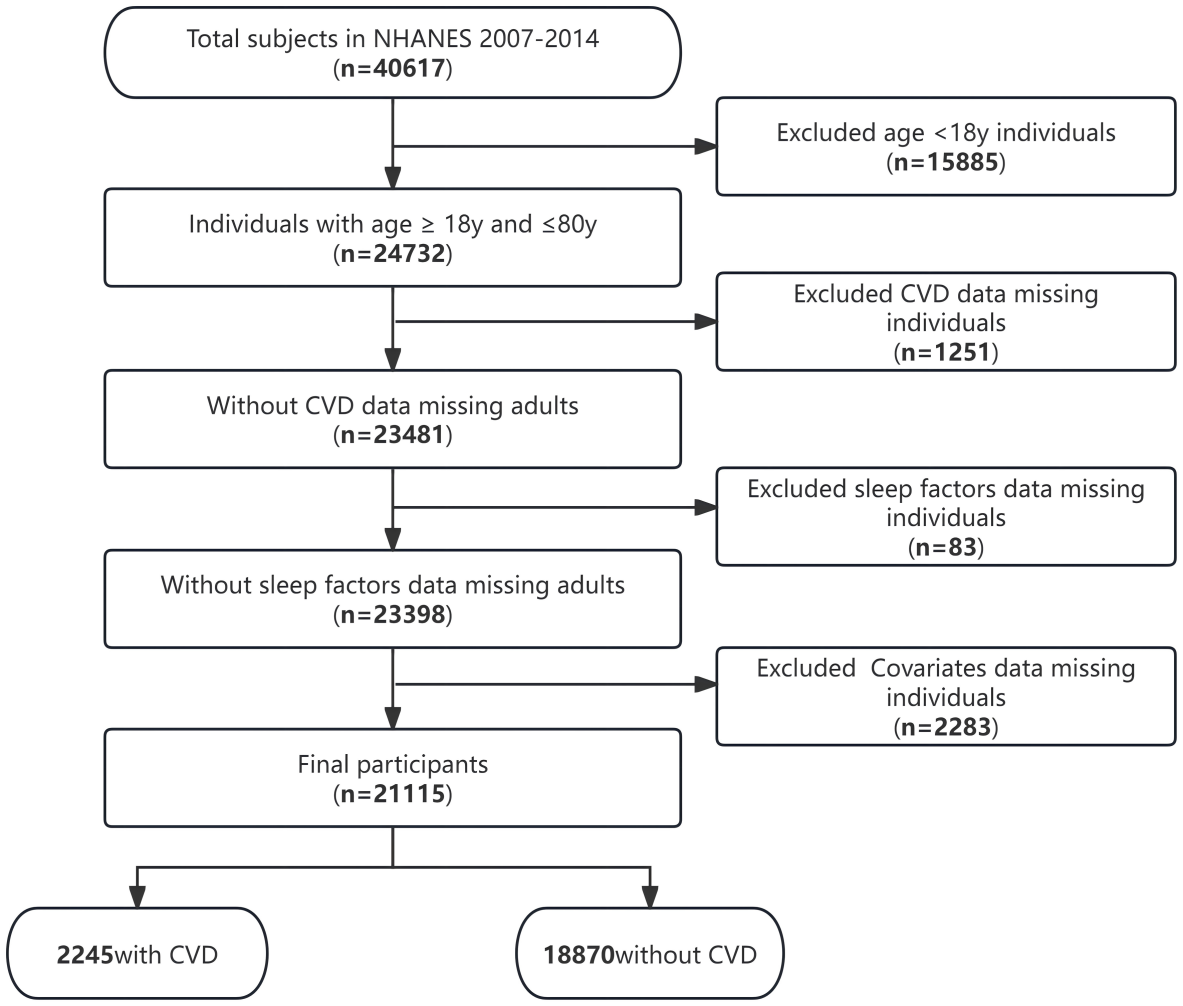
Flow chart of the study population inclusion.

The present research encompassed 21,115 individuals who were subjected to an extensive screening procedure guided by predetermined inclusive and exclusive criteria (Figure 1). Among these individuals, there was an overall 10.6% prevalence of CVD.

Table 1 displays the baseline characteristics of the participants in the study, which were categorized according to their sleep patterns. According to research, individuals with disrupted sleep patterns seem to have a greater likelihood of developing CVD. Among the 21,115 participants, 48.2% were male, with an average age (SD) of 47.2 (16.8) years. According to the data, 42.5% of individuals exhibited healthy sleep patterns, while 39.3 and 18.2% had intermediate and poor sleep patterns, respectively. It was observed that individuals with healthy sleep patterns tended to be younger, have a lower BMI, and were more likely to be male, married, more physically active (vigorous), and non-smokers. There were no statistically significant differences in education level, drinking habit, and TC subgroups.

**Table 1:** Study sample characteristics by sleep pattern status.

Variables	Sleep patterns (Col %)				
	Total (*n* = 21,115)	Healthy (*n* = 8,979)	Intermediate (*n* = 8,287)	Poor (*n* = 3,849)	*p*
Age(y), Mean ± SD	47.2 ± 16.8	46.3 ± 17.1	46.7 ± 16.8	50.5 ± 15.6	<0.001
*Sex, *n* (%)					<0.001
Male	10,276 (48.2)	4,532 (50.1)	4,076 (48.3)	1,668 (43.8)	
Female	10,839 (51.8)	4,447 (49.9)	4,211 (51.7)	2,181 (56.2)	
*Race, *n* (%)					<0.001
Mexican American	3,172 (8.5)	1,571 (9.9)	1,211 (8.4)	390 (5.3)	
Other Hispanic	2,141 (5.5)	949 (5.8)	822 (5.6)	370 (4.6)	
Non-Hispanic white	9,430 (68.1)	3,987 (68.3)	3,460 (65.4)	1,983 (73.4)	
Non-Hispanic black	4,260 (10.7)	1,465 (8.3)	1,944 (12.9)	851 (11.7)	
Others	2,112 (7.2)	1,007 (7.6)	850 (7.6)	255 (5.1)	
*Marital status, *n* (%)					<0.001
Married	10,949 (56.0)	4,999 (59.8)	4,135 (54.1)	1,815 (51)	
Never married	3,846 (18.1)	1,656 (18.4)	1,613 (19.2)	577 (15.2)	
Others	6,320 (25.9)	2,324 (21.8)	2,539 (26.8)	1,457 (33.8)	
*Education level, *n* (%)					0.808
<9	2,284 (5.8)	1,025 (6.1)	876 (5.6)	383 (5.5)	
9–12	3,197 (11.7)	1,332 (11.1)	1,282 (12.5)	583 (11.7)	
>12	15,634 (82.4)	6,622 (82.7)	6,129 (81.9)	2,883 (82.9)	
*Insurance, *n* (%)					<0.001
No insurance	4,978 (19.3)	2,369 (20.4)	2,008 (20.4)	601 (14.2)	
Any insurance	16,137 (80.7)	6,610 (79.6)	6,279 (79.6)	3,248 (85.8)	
**BMI (kg/m2), Mean ± SD**	28.8 ± 6.7	28.0 ± 6.2	28.7 ± 6.5	30.9 ± 8.0	<0.001
*Hypertension, *n* (%)					<0.001
No	13,564 (68.3)	6,314 (73.7)	5,448 (69.7)	1,802 (52.2)	
Yes	7,551 (31.7)	2,665 (26.3)	2,839 (30.3)	2,047 (47.8)	
*Diabetes mellitus, *n* (%)					<0.001
No	18,069 (88.9)	7,946 (91.6)	7,191 (89.9)	2,932 (80.6)	
Borderline	464 (2.1)	169 (1.8)	150 (1.5)	145 (3.7)	
Yes	2,582 (9.0)	864 (6.6)	946 (8.5)	772 (15.7)	
*Smoking status, *n* (%)					<0.001
Never	11,592 (55.2)	5,349 (60.6)	4,501 (53.9)	1,742 (44.8)	
Former	5,082 (24.3)	2,076 (23.4)	1,940 (23.4)	1,066 (28.4)	
Current	4,441 (20.5)	1,554 (16.0)	1,846 (22.6)	1,041 (26.8)	
*Drinking habit, *n* (%)					0.718
No	5,293 (20.6)	2,275 (20.4)	2,066 (20.9)	952 (20.6)	
Yes	15,822 (79.4)	6,704 (79.6)	6,221 (79.1)	2,897 (79.4)	
*Physical activity, *n* (%)					<0.001
Sedentary	11,212 (47.1)	4,491 (42.9)	4,432 (47.9)	2,289 (55.5)	
Moderate	5,585 (28.5)	2,475 (29.8)	2,115 (27.9)	995 (26.9)	
Vigorous	4,318 (24.4)	2,013 (27.4)	1,740 (24.2)	565 (17.6)	
**HbA1C (%), Mean ± SD**	5.6 ± 0.9	5.6 ± 0.8	5.6 ± 0.9	5.8 ± 1.0	<0.001
**TC (mg/dl), Mean ± SD**	194.5 ± 41.5	194.6 ± 40.5	194.7 ± 42.1	193.9 ± 42.5	0.704
**HDL-C (mg/dl), Mean ± SD**	52.8 ± 16.1	53.4 ± 16.0	52.8 ± 15.8	51.5 ± 16.5	0.001
*CVD, *n* (%)					<0.001
No	18,870 (91.6)	8,258 (93.5)	7,492 (92.7)	3,120 (95.1)	
Yes	2,245 (8.4)	721 (6.5)	795 (7.3)	729 (4.9)	
*CHF, *n* (%)					<0.001
No	20,451 (97.5)	8,804 (98.4)	8,037 (97.5)	3,610 (93.8)	
Yes	664 (2.5)	175 (1.6)	250 (2.5)	239 (6.2)	
*CHD, *n* (%)					<0.001
No	20,280 (96.5)	8,680 (96.9)	8,002 (97.2)	3,598 (94.0)	
Yes	835 (3.5)	299 (3.1)	285 (2.8)	251 (6.0)	
*Angina, *n* (%)					<0.001
No	20,601 (97.8)	8,839 (98.5)	8,113 (98.2)	3,649 (95.5)	
Yes	514 (2.2)	140 (1.5)	174 (1.8)	200 (4.5)	
*MI, *n* (%)					<0.001
No	20,241 (96.7)	8,707 (97.4)	7,985 (97.3)	3,549 (93.7)	
Yes	874 (3.3)	272 (2.6)	302 (2.7)	300 (6.3)	
*Stroke, *n* (%)					<0.001
No	20,332 (97.1)	8,734 (97.8)	8,007 (97.5)	3,591 (94.4)	
Yes	783 (2.9)	245 (2.2)	280 (2.5)	258 (5.6)	
*Trouble sleeping, *n* (%)					<0.001
No	15,905 (73.8)	8,979 (100.0)	6,753 (76.7)	173 (4.5)	
Yes	5,210 (26.2)	0 (0.0)	1,534 (23.3)	3,676 (95.5)	
*Sleep disorder, *n* (%)					<0.001
No	19,322 (91.3)	8,979 (100.0)	8,116 (97.6)	2,227 (57.3)	
Yes	1,793 (8.7)	0 (0.0)	171 (2.4)	1,622 (42.7)	
*Sleep duration (hours), *n* (%)					<0.001
7–9	11,201 (53.0)	8,979 (100.0)	1,705 (20.6)	517 (13.4)	
<7	8,315 (39.4)	0 (0.0)	5,330 (64.3)	2,985 (77.6)	
>9	1,599 (7.6)	0 (0.0)	1,252 (15.1)	347 (9.0)	

TC, Total cholesterol; HDL-C, High density lipoprotein cholesterol; HbA1C, Glycosylated hemoglobin; CVD, Cardiovascular diseases; CHF, congestive heart failure; CHD, coronary heart disease; MI, myocardial infarction.

^*^Data presented as unweighted numbers (weighted percentage) for categorical variables and mean (standard error) for continuous variables.

### 3.2 Relationship between sleep and CVD

Univariate analysis showed that all covariates, except race, were associated with CVD (Supplementary Table S1).

Upon categorizing sleep patterns into three levels, healthy, intermediate, and poor, we observed a noteworthy positive correlation between sleep patterns and CVD, even after accounting for potential confounding factors. In comparison to those with a healthy sleep pattern, the adjusted OR for CVD risk in individuals with intermediate and poor sleep patterns were 1.03 (95% CI: 0.84 ~ 1.25, *p* = 0.787) and 1.82 (95% CI: 1.52 ~ 2.16, *p* < 0.001), respectively (Table 2).

**Table 2:** Weighted odds ratios with 95% CI for the associations between sleep patterns and CVD.

Quartiles	OR (95% CI)								
	NO.	Crude	*p*-value	Model 1	*p*-value	Model 2	*p*-value	Model 3	*p*-value
**Sleep patterns**									
Healthy	8,979	1 (Ref)		1 (Ref)		1 (Ref)		1 (Ref)	
Intermediate	8,287	1.12 (1.01 ~ 1.25)	0.028	1.26 (0.95 ~ 1.41)	<0.132	1.12 (0.92 ~ 1.36)	0.258	1.03 (0.84 ~ 1.25)	0.787
Poor	3,849	2.63 (2.27 ~ 3.04)	<0.001	2.56 (2.18 ~ 3.00)	<0.001	2.49 (2.11 ~ 2.93)	<0.001	1.82 (1.52 ~ 2.16)	<0.001
Trend test	21,115		<0.001		<0.001		<0.001		<0.001

Q, quartiles; OR, odds ratio; CI, confidence interval; Ref: reference.

Model 1 was adjusted for age, sex. Model 2 was adjusted for adjusted for age, sex, race, marital status, education level, and health insurance. Model 3 was adjusted for age, sex, race, marital status, education level, health insurance, BMI, hypertension, diabetes mellitus, smoking status, drinking habit, physical activity, HbA1C, TC, and HDL-C.

**Figure 2:**
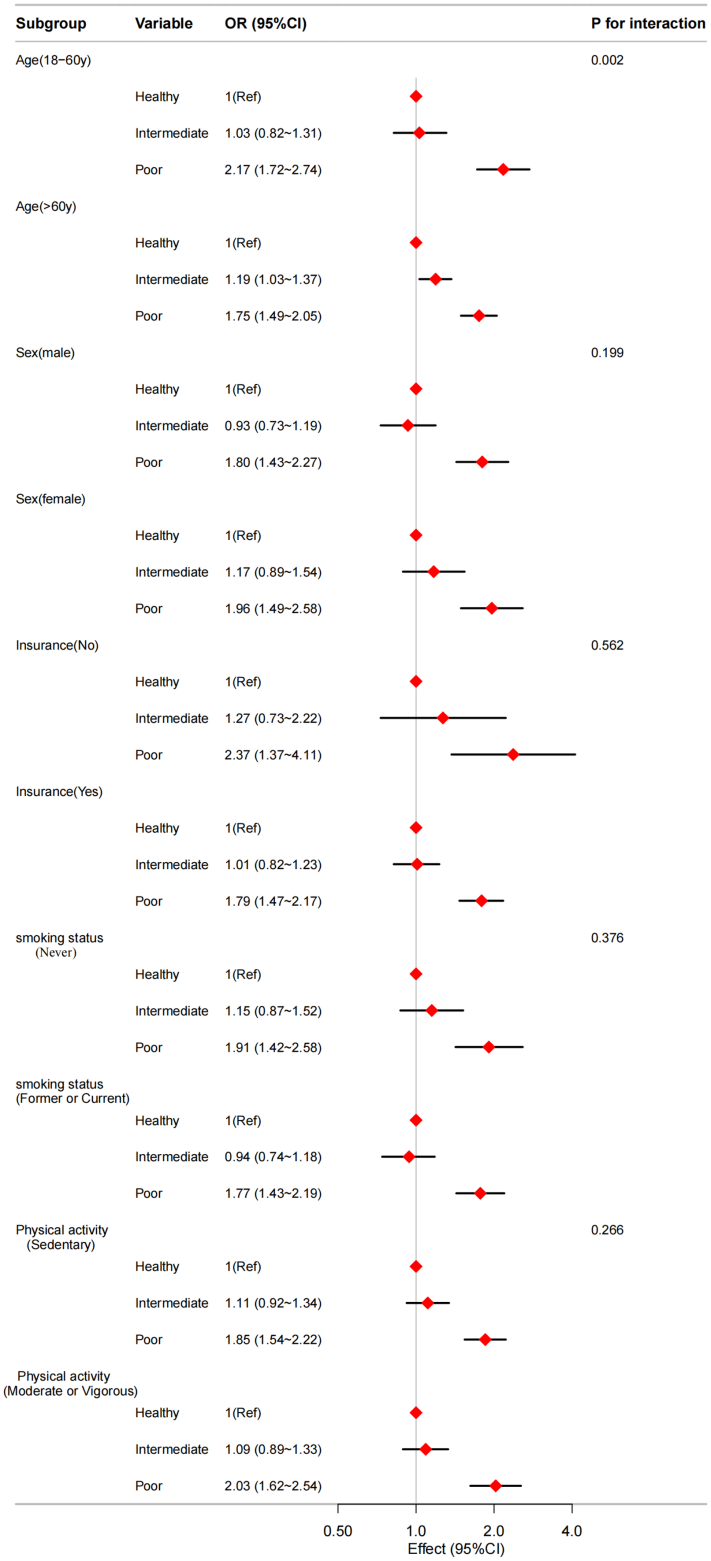
The relationship between sleep patterns and CVD according to basic features. Except for the stratification component itself, each stratification factor was adjusted for all other variables (age, sex, race, marital status, education level, health insurance, BMI, hypertension, diabetes mellitus, smoking status, drinking habit, physical activity, HbA1C, TC, and HDL-C).

To assess potential modifications in the connection between sleep patterns and CVD, stratified analyses were conducted in multiple subgroups, including the variables: age (20–60y vs. >60y), sex (male vs. female), insurance (no vs. yes), smoking status (never vs. former or current), and physical activity (sedentary vs. moderate or vigorous). There is a significant interaction between sleep patterns and age (P for interaction = 0.002) shown in Figure 2.

We conducted multivariable analysis to examine the associations between each of the three elements (sleep duration, self-reported trouble sleeping, and diagnosed sleep disorder) within sleep patterns and CVD. In sleep duration, compared with individuals with normal sleep duration, the adjusted OR values in short and long sleep duration were 1.12 (95%CI: 0.95–1.33, *p* = 0.174), and 1.14 (95%CI:0.90–1.45, *p* = 0.266) (Supplementary Table S2). In self-reported trouble sleeping, compared with individuals with no trouble sleeping, the adjusted OR values in trouble sleeping were 1.53 (95%CI: 1.32–1.78, *p* < 0.001) (Supplementary Table S3). In diagnosed sleep disorder, compared with individuals with no sleep disorder, the adjusted OR values in sleep disorder were 2.09 (95%CI: 1.75–2.50, *p* < 0.001) (Supplementary Table S4). Additionally, a significant U-shaped curve (nonlinear, *p* < 0.001) was observed in the correlation between sleep duration and CVD in the restricted cubic spline (RCS) analysis (Supplementary Figure S1).

As shown in Supplementary Table S5, following the adjustment for all covariates within our full model (Model 3), it was found that poor sleep patterns exhibited a positive correlation with all subtypes of CVD risk in comparison to healthy sleep patterns (all *p* < 0.05).

## 4 Discussion

In this nationally representative study, a clear correlation was found between sleep factors and increased risk of cardiovascular disease. Moreover, a positive dose–response relationship was observed, indicating that the risk of CVD increased with poorer sleep patterns. Intermediate and poor sleep patterns were associated with a 3 and 82% higher incidence of CVD, respectively, compared to participants with healthy sleep patterns.

There’s some debate about the relationship between sleep duration and the risk of cardiovascular diseases. In three cohort studies (Wang et al., 2016; Yang et al., 2016; Song et al., 2017) involving Chinese populations, both cardiovascular diseases (CHD and atrial fibrillation) had been reported to have a relationship with variations in sleep duration. Similarly, sleep durations that are both short and long have been connected with increased risks of stroke morbidity and mortality (Brunetti et al., 2022). However, it is worth noting that individuals who sleep for extended periods are more likely to experience negative consequences than those who sleep for shorter durations (Wang et al., 2022). In a cross-sectional (Chen et al., 2022) study utilizing the NHANES database, it was discovered that sleep duration exhibited a significant relationship with an increased incidence of chest pain. Both short and long sleep durations were found to heighten the risk of chest pain, while the optimal quantity of sleep to mitigate this risk was approximately 6.5 h. However, a few studies have reported inconsistent results. In a study of the British population by Wannamethee et al. (2016), only patients with short sleep durations were found to be positively associated with the risk of hypertension, coronary artery calcification, and heart failure. A meta-analysis (Cappuccio et al., 2011) found that only long sleep duration was positively associated with the risk of CVD. In a study conducted by Song et al. (2016) there was no reported association between myocardial infarction and abnormal sleep duration. In our study, neither short nor long sleep durations were positively correlated with CVD risk. These discrepancies may be attributed to the inconsistent definitions of normal sleep duration, as well as the varying methods used to assess sleep duration and the diversity of study populations. This underscores the need for further research to establish the optimal sleep duration and to standardize the measurement methods for sleep duration.

Wang et al.’s study (Wang et al., 2020) did not identify an association between sleep duration and CVD, however, a comprehensive examination of the relationship between short sleep duration, insomnia/poor sleep, and cardiovascular disease did reveal such an association. In several previous studies (Bertisch et al., 2018; Chandola et al., 2010; Chien et al., 2010) on combinations of sleep factors, it was found that participants with short or long sleep durations, frequent symptoms of insomnia, as well as snoring and daytime sleepiness, had the highest risk of cardiovascular disease and were associated with all-cause mortality. This finding suggests that, in addition to sleep duration, altered sleep quality (insomnia/poor sleep) may also influence CVD. Hence, a composite of sleep factors may offer a more precise depiction of this relationship. In light of this, we introduced a novel metric, the healthy sleep score, which considers the combined impacts of three sleep behaviors (Li and Shang, 2021) (sleep duration, sleep disorders, and trouble sleeping) on the risk of CVD. This score captures a more holistic view of the sleep patterns. The healthy sleep pattern, as defined in our study (7–9 h of sleep per day, absence of sleep disorders and no reported trouble sleeping), establishes a favorable benchmark for sleep, serving as a useful reference to identify populations at risk and promote health management. From a public health standpoint, a straightforward scoring algorithm enhances the interpretability of epidemiological findings, facilitating their translation into practical insights for the general population. In an observational study by Fan et al. (2020), which also used sleep scores to assess their effect on CVD, it was concluded that healthy sleep patterns reduced the risk of CVD, CHD, and stroke in participants; however, this study was only conducted on a European population, and our study complements the U.S. population.

The results of our subgroup analyses revealed an interaction between sleep patterns and CVD risk across different age groups, indicating that the impact of sleep patterns on cardiovascular health varies by age. Among individuals under 60 years of age, poorer sleep patterns were linked to a significantly increased risk of CVD. This finding aligns with existing literature (Cappuccio et al., 2011), suggesting that inadequate sleep habits can adversely affect cardiovascular health even in younger adults. The relationship between sleep patterns and CVD risk appears to become more complex with advancing age. In individuals aged 60 and older, poorer sleep patterns and intermediate sleep patterns were associated with an increased risk of CVD, potentially reflecting the cumulative effects of chronic sleep disturbances on the cardiovascular system (Laksono et al., 2022).

The various mechanisms underlying the relationship between sleep and the onset of CVD are remain unclear at present. These Include dysregulation of the hypothalamus-pituitary–adrenal (HPA) axis (Grandner et al., 2016), abnormalities in autonomic nervous system regulation (Johnson et al., 2021), increased sympathetic nervous system (SNS) activity (Parthasarathy et al., 2015; Seravalle et al., 2018), insulin resistance (Kline et al., 2018), changes in inflammatory and hormonal markers (due to increased secretion of pro-inflammatory cytokines or an inverse correlation with the ratio of vascular collagen elastin proteins) (D'Antono and Bouchard, 2019), and impaired endothelial function (De Nys et al., 2022). Hong et al. (2023) found that fibrinogen may be a mechanism for the development of CHD in participants with long sleep duration, although the exact mechanism is unknown, and may be related to depressive symptoms, low socioeconomic status, unemployment, and low physical activity. A meta-analysis (Irwin et al., 2016) conducted on cohort studies discovered that sleep disruption and extended sleep durations (as opposed to insufficient sleep) were linked to increased levels of systemic inflammatory biomarkers such as CRP and IL-6, which may contribute to the higher incidence of CVD. Several biological mechanisms have been suggested for sleep disorders that can affect the regulation of the central nervous system (Tamisier et al., 2018), hemodynamics (Yang et al., 2019), ventilatory function (Chowdhuri and Badr, 2017), and biorhythms (Makarem et al., 2019), leading to altered cardiac physiology and pathological changes in blood pressure.

In addition to sleep patterns, dietary habits represent another critical factor influencing CVD risk. Specifically, a diet abundant in fruits, vegetables, whole grains, nuts, and fish, while limiting the intake of processed foods, as well as foods high in salt and sugar, has been demonstrated to lower CVD risk (Ghaedi et al., 2019). Moreover, the Mediterranean dietary pattern, characterized by high olive oil consumption and moderate alcohol intake, is linked to reduced morbidity and mortality associated with cardiovascular diseases (Estruch et al., 2018). Therefore, future research should aim to incorporate dietary factors into their analyses to facilitate a more comprehensive assessment of cardiovascular disease risk.

The study boasts several strengths, notably the utilization of a substantial and nationally representative sample from the NHANES 2007–2014 database, offering an extensive array of potential confounders for analysis. In addition, by consolidating sleep quality and quantity (including sleep duration, self-reported sleep difficulties, and sleep disorders) into a unified sleep metric, we comprehensively address the intricate nature of sleep. Nevertheless, certain limitations are acknowledged in this study. Firstly, given its cross-sectional design, causal relationships between sleep and CVD cannot be established. Secondly, reliance on self-reported sleep data introduces potential memory bias, and sleep duration information is confined to weekdays, omitting details on shift work and weekend sleep duration. Ambiguities in the categorization of self-reported sleep disorders preclude a precise examination of their association with CVD. Thirdly, despite employing regression models and stratification analyses, we cannot exclude the possibility that the observed associations are due to unmeasured confounders. Fourthly, the current findings stem from surveys conducted among U.S. adults, and further research is needed to determine generalizability to other populations. It is imperative to recognize the observational nature of this study, warranting cautious interpretations of the results. Future clinical trials on sleep interventions are imperative to discern the causal nature of the observed associations.

## 5 Conclusion

Participants who exhibit poor sleep patterns have been found to be at a significantly higher risk for developing CVD. Sleep, as a modifiable behavioral factor, plays a crucial role in the prevention of primary cardiovascular disease. To establish a causal relationship between sleep and cardiovascular disease, further prospective studies are necessary.

## Data Availability

The original contributions presented in the study are included in the article/Supplementary material, further inquiries can be directed to the corresponding author.

## Supplementary material

SUPPLEMENTARY FIGURE S1 Non-linear relationship of sleep duration and CVD. Solid and dashed lines represent the predicted value and 95% confidence intervals. They were adjusted for age, sex, race, marital status, education level, health insurance, BMI, hypertension, diabetes mellitus, smoking status, drinking habit, physical activity, HbA1C, TC, and HDL-C. All of the data is shown.
