# Supplementary figures and images for: Sleep patterns and cardiovascular disease risk in US participants: a comprehensive analysis
Source: Front Neurosci. 2025 Jan 9;18:1447543. doi: 10.3389/fnins.2024.1447543 (PMC11754222; doi:10.3389/fnins.2024.1447543)

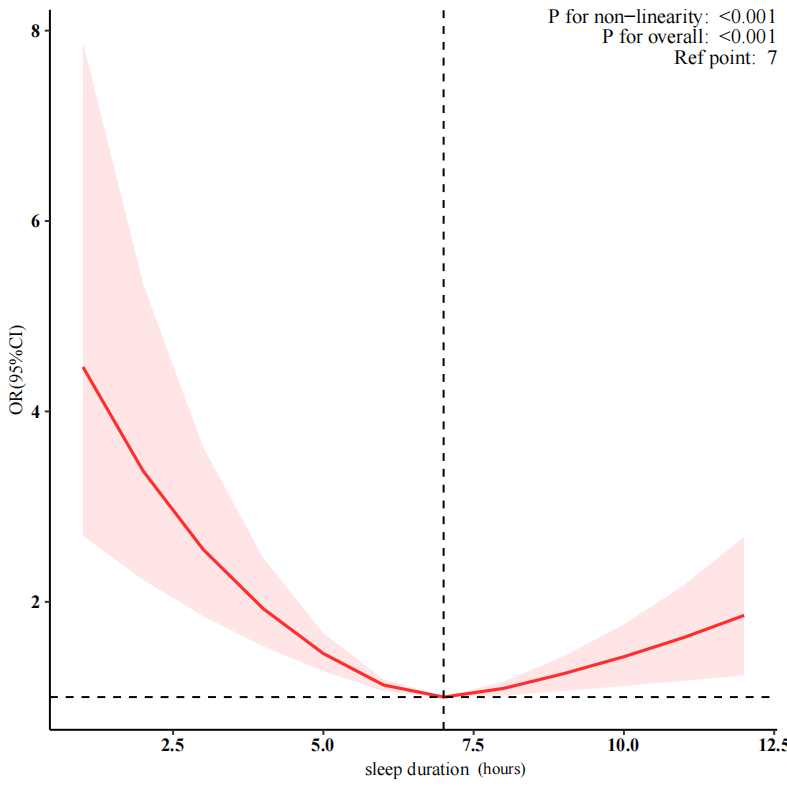

Supplement: SUPPLEMENTARY FIGURE S1 — Non-linear relationship of sleep duration and CVD. Solid and dashed lines represent the predicted value and 95% confidence intervals. They were adjusted for age, sex, race, marital status, education level, health insurance, BMI, hypertension, diabetes mellitus, smoking status, drinking habit, physical activity, HbA1C, TC, and HDL-C. All of the data is shown. [file Image_1.TIF]
