# Supplementary material for: Sleep patterns and cardiovascular disease risk in US participants: a comprehensive analysis
Source: Front Neurosci. 2025 Jan 9;18:1447543. doi: 10.3389/fnins.2024.1447543 (PMC11754222; doi:10.3389/fnins.2024.1447543)
Supplement: Supplementary file 2 [file Data_Sheet_1.docx]

**Table S1**. Association of covariates and CVD.

| Variable | OR_95CI | P_ value |
| --- | --- | --- |
| **Age（y）** | 1.08 (1.07~1.08) | <0.001 |
| **Gender，n%** |  |  |
| Male | 1 (reference) |  |
| Female | 0.71 (0.65~0.77) | <0.001 |
| **Race, n (%)** |  |  |
| Mexican American | 1 (reference) |  |
| Other Hispanic | 1.14 (0.92~1.41) | 0.221 |
| Non-Hispanic white | 2.14 (1.84~2.49) | <0.001 |
| Non-Hispanic black | 1.69 (1.42~1.99) | <0.001 |
| Others | 0.92 (0.73~1.15) | 0.448 |
| **Marital status, n (%)** |  |  |
| Married | 1 (reference) |  |
| Never married | 0.39 (0.33~0.46) | <0.001 |
| Others | 1.45 (1.32~1.59) | <0.001 |
| **Education level, n (%)** |  |  |
| <9 | 1 (reference) |  |
| 9–12 | 0.79 (0.68~0.92) | 0.002 |
| >12 | 0.53 (0.47~0.6) | <0.001 |
| **Insurance, n (%)** |  |  |
| No insurance | 1 (reference) |  |
| Any insurance | 3.24 (2.8~3.75) | <0.001 |
| **BMI (kg/m2)** | 1.03 (1.02~1.04) | <0.001 |
| **Hypertension, n (%)** |  |  |
| No | 1 (reference) |  |
| Yes | 6.67 (6.03~7.38) | <0.001 |
| **Diabetes mellitus, n (%)** |  |  |
| No | 1 (reference) |  |
| Borderline | 3.08 (2.45~3.88) | <0.001 |
| Yes | 4.52 (4.08~5) | <0.001 |
| **Smoking, n (%)** |  |  |
| Never | 1 (reference) |  |
| Former | 2.51 (2.27~2.77) | <0.001 |
| Current | 1.45 (1.29~1.63) | <0.001 |
| **Physical activity, n (%)** |  |  |
| Sedentary | 1 (reference) |  |
| Moderate | 0.7 (0.63~0.78) | <0.001 |
| Vigorous | 0.2 (0.17~0.24) | <0.001 |
| **HbA1C (%)** | 1.39 (1.35~1.43) | <0.001 |
| **TC (mg/dl)** | 0.99 (0.99~0.99) | <0.001 |
| **HDL-C(mg/dl)** | 0.99 (0.98~0.99) | <0.001 |
| **Sleep duration (hours)** |  |  |
| 7-9 | 1 (reference) |  |
| <7 | 1.16 (1.05~1.27) | 0.002 |
| >9 | 1.94 (1.68~2.25) | <0.001 |
| **Trouble sleeping** |  |  |
| No | 1 (reference) |  |
| Yes | 2.2 (2.01~2.41) | <0.001 |

**Table S1**(Continued)

| Variable | OR_95CI | P_ value |
| --- | --- | --- |
| **Sleep disorder** |  |  |
| No | 1 (reference) |  |
| Yes | 2.81 (2.5~3.17) | <0.001 |
| **Sleep patterns** |  |  |
| Healthy | 1 (reference) |  |
| Intermediate | 1.22 (1.09~1.35) | <0.001 |
| Poor | 2.68 (2.4~2.99) | <0.001 |

TC: Total cholesterol; HDL-C: High density lipoprotein cholesterol; HbA1C: Glycosylated hemoglobin; CVD: Cardiovascular diseases; CHF: congestive heart failure; CHD: coronary heart disease; MI: myocardial infarction.

**Table S2**. Weighted odds ratios with 95% CI for the associations between sleep duration and CVD.

| **Quartiles** | **OR (95% CI)** | | | | | | | | |
| --- | --- | --- | --- | --- | --- | --- | --- | --- | --- |
|  | **NO.** | **Crude** | **p-Value** | **Model1** | **p-Value** | **Model2** | **p-Value** | **Model3** | **p-Value** |
| Hours of Sleep |  |  |  |  |  |  |  |  |  |
| <7 | 8315 | 1.11 (0.96~1.28) | 0.147 | 1.35 (1.16~1.57) | <0.001 | 1.29 (1.09~1.51) | 0.003 | 1.12 (0.95~1.33) | 0.174 |
| 7-9 | 11201 | 1(Ref) |  | 1(Ref) |  | 1(Ref) |  | 1(Ref) |  |
| ≥9 | 1599 | 1.81 (1.48~2.20) | <0.001 | 1.33  (1.08~1.64) | 0.008 | 1.28 (1.04~1.57) | 0.019 | 1.14 (0.90~1.45) | 0.266 |
| Trend test | 21115 |  | <0.001 |  | <0.001 |  | 0.001 |  | 0.126 |

Q, quartiles; OR, odds ratio; CI, confidence interval; Ref: reference.

**Model 1** was adjusted for age, sex. **Model 2** was adjusted for adjusted for age, sex, race, marital status, education level, and health insurance. **Model 3** was adjusted for age, sex, race, marital status, education level, health insurance, BMI, hypertension, diabetes mellitus, smoking status, drinking habit, physical activity, HbA1C, TC, and HDL-C.

**Table S3**. Weighted odds ratios with 95% CI for the associations between trouble sleeping and CVD.

| **Quartiles** | **OR (95% CI)** | | | | | | | | |
| --- | --- | --- | --- | --- | --- | --- | --- | --- | --- |
|  | **NO.** | **Crude** | **p-Value** | **Model1** | **p-Value** | **Model2** | **p-Value** | **Model3** | **p-Value** |
| Trouble sleeping |  |  |  |  |  |  |  |  |  |
| No | 15905 | 1(Ref) |  | 1(Ref) |  | 1(Ref) |  | 1(Ref) |  |
| Yes | 5210 | 1,98 (1.76~2.24) | <0.001 | 1.87 (1.64~2.14) | <0.001 | 1.87 (1.63~2.14) | <0.001 | 1.53 (1.32~1.78) | <0.001 |

Q, quartiles; OR, odds ratio; CI, confidence interval; Ref: reference.

**Model 1** was adjusted for age, sex. **Model 2** was adjusted for adjusted for age, sex, race, marital status, education level, and health insurance. **Model 3** was adjusted for age, sex, race, marital status, education level, health insurance, BMI, hypertension, diabetes mellitus, smoking status, drinking habit, physical activity, HbA1C, TC, and HDL-C.

**Table S4**. Weighted odds ratios with 95% CI for the associations between sleep disorder and CVD.

| **Quartiles** | **OR (95% CI)** | | | | | | | | |
| --- | --- | --- | --- | --- | --- | --- | --- | --- | --- |
|  | **NO.** | **Crude** | **p-Value** | **Model1** | **p-Value** | **Model2** | **p-Value** | **Model3** | **p-Value** |
| sleep disorder |  |  |  |  |  |  |  |  |  |
| No | 19322 | 1(Ref) |  | 1(Ref) |  | 1(Ref) |  | 1(Ref) |  |
| Yes | 1793 | 3.06 (2.66~3.53) | <0.001 | 2.90 (2.46~3.42) | <0.001 | 2.93 (2.48~3.46) | <0.001 | 2.09 (1.75~2.50) | <0.001 |

Q, quartiles; OR, odds ratio; CI, confidence interval; Ref: reference.

**Model 1** was adjusted for age, sex. **Model 2** was adjusted for adjusted for age, sex, race, marital status, education level, and health insurance. **Model 3** was adjusted for age, sex, race, marital status, education level, health insurance, BMI, hypertension, diabetes mellitus, smoking status, drinking habit, physical activity, HbA1C, TC, and HDL-C.

**Table S5.** Multivariable weighted odds ratios with 95% CI for the association between sleep patterns and risk of CVD subtypes.

| **Characteristics** | **Healthy** | **Intermediate** | **p-Value** | **Poor** | **p-Value** | **P for trend** |
| --- | --- | --- | --- | --- | --- | --- |
| **CHF** | 1(Ref) | 1.48 (1.13~1.94) | <0.001 | 1.99 (1.53~2.59) | <0.001 | <0.001 |
| **CHD** | 1(Ref) | 0.88 (0.67~1.15) | 0.325 | 1.36 (1.02~1.79) | 0.035 | 0.077 |
| **angina** | 1(Ref) | 1.17 (0.80~1.69) | 0.412 | 2.02 (1.46~2.81) | <0.001 | <0.001 |
| **MI** | 1(Ref) | 1.01 (0.81~1.25) | 0.982 | 1.69 (1.29~2.21) | <0.001 | <0.001 |
| **stroke** | 1(Ref) | 0.93 (0.71~1.21) | 0.567 | 1.70 (1.28~2.24) | <0.001 | 0.001 |

CHF: congestive heart failure; CHD: coronary heart disease; MI: myocardial infarction.

*Adjusted for age, sex, race, marital status, education level, health insurance, BMI, hypertension, diabetes mellitus, smoking status, drinking habit, physical activity, HbA1C, TC, and HDL-C.
